# The molecular mechanisms of MLKL-dependent and MLKL-independent necrosis

**DOI:** 10.1093/jmcb/mjaa055

**Published:** 2020-10-16

**Authors:** Lu Li, An Tong, Qiangsheng Zhang, Yuquan Wei, Xiawei Wei

**Affiliations:** 1 Laboratory of Aging Research and Cancer Drug Target, State Key Laboratory of Biotherapy and Cancer Center, National Clinical Research Center for Geriatrics, West China Hospital, Sichuan University, Chengdu 610041, China; 2 Department of Gynecology and Obstetrics, Development and Related Disease of Women and Children Key Laboratory of Sichuan Province, Key Laboratory of Birth Defects and Related Diseases of Women and Children, Ministry of Education, West China Second Hospital, and State Key Laboratory of Biotherapy, West China Hospital, Sichuan University, Chengdu 610041, China

**Keywords:** MLKL, necrosis, necroptosis, mechanism, diseases

## Abstract

Necrosis, a type of unwanted and passive cell demise, usually occurs under the excessive external stress and is considered to be unregulated. However, under some special conditions such as caspase inhibition, necrosis is regulable in a well-orchestrated way. The term ‘regulated necrosis’ has been proposed to describe such programed necrosis. Recently, several forms of necrosis, including necroptosis, pyroptosis, ferroptosis, parthanatos, oxytosis, NETosis, and Na^+^/K^+^-ATPase-mediated necrosis, have been identified, and some crucial regulators governing regulated necrosis have also been discovered. Mixed lineage kinase domain-like pseudokinase (MLKL), a core regulator in necroptosis, acts as an executioner in response to ligands of death receptor family. Its activation requires the receptor-interacting protein kinases, RIP1 and RIP3. However, MLKL is only involved in necroptosis, i.e. MLKL is dispensable for necrosis. Therefore, this review is aimed at summarizing the molecular mechanisms of MLKL-dependent and MLKL-independent necrosis.

## Introduction

Cell death is a crucial method for organisms to maintain tissue homeostasis by eliminating abnormal, misplaced, nonfunctional, and harmful cells. In general, there are mainly three types of morphologically distinct cell death: apoptosis, autophagic cell death, and necrosis (Galluzzi et al., 2007). Necrosis is always considered to occur in accidental settings under some extreme microenvironmental conditions (e.g. elevated pressure, shear force, and high temperature). However, the discovery of the regulated necrosis, which can be modulated by pharmacological agents or genetic interventions, has greatly changed this view. In the past decades, several forms of regulated necrosis have been identified, including necroptosis (Degterev et al., 2005), pyroptosis (Mariathasan et al., 2004), ferroptosis (Yang and Stockwell, 2008), parthanatos (Andrabi et al., 2008), oxytosis (Tan et al., 2001), and NETosis (Yipp et al., 2012).

Necroptosis, a well-characterized regulated necrosis, is receptor-interacting protein (RIP) kinase-dependent cell death with the morphological features of necrosis (He et al., 2009). The molecular mechanism of necroptosis has been under intensive investigation in recent years. Mixed lineage kinase domain-like pseudokinase (MLKL) is considered to be the key mediator of necroptosis. After necroptosis initiation, MLKL together with RIP1 and RIP3 forms necrosome, and the phosphorylated MLKL homo-oligomerizes and translocates to the plasma membrane (PM) to induce cell death. However, how MLKL oligomers trigger cell death is still under hot debate. Interestingly, increasing evidence suggested that MLKL is not integrant in all necroptosis, and there are some other potential downstream proteins or pathways to mediate necroptosis. Thus, it is necessary to figure out the mechanism of necroptosis and other types of regulated necrosis in which MLKL is involved or not.

Plenty of evidence suggests that necrosis is a double-edged sword. It is not only associated with pathophysiology of some diseases (Galluzzi et al., 2017) but also activates innate immunity in response to tissue injury or viral infection (Gaiha et al., 2014). Favorably, necrosis induces an innate immune response to protect the body from virus infection or other injuries. On the other hand, necrosis *per se* is harmful, causing various diseases such as tissue sepsis, hepatic disorders (Rosentreter et al., 2015), renal damage (Xu et al., 2015), neurodegenerative diseases (Degterev et al., 2005), and cardiovascular diseases (Oerlemans et al., 2012).

## Necrosis and necroptosis

The term ‘necrosis’, which originates from the Greek word ‘nekros’, has been used for ∼2000 years to describe drastic tissue changes visible to the naked eye formerly (Majno and Joris, 1995). Traditionally, necrosis is regarded as an accidental and irreparable cell death, and it generally occurs as a consequence of various physical and physiological stimuli, such as high temperature, freeze‒thaw, mechanical stress, irradiation, ischemia, pathogens, and cytokines (Proskuryakov et al., 2003). Morphologically, cell and organelles swell and break down with the subsequent release of cellular components into the microenvironment. Contrary to the traditional belief, multiple types of regulated necrosis are emerging, and the features of these regulated necrosis are described and summarized in Table 1.

**Table 1:** The brief comparison of several types of necrosis.

Items	Biological features	Stimuli	Key molecules	Inhibitors
Necroptosis	RIP1/RIP3/MLKL activation	TNF, Fas, TRAIL ligand, dsRNA, dsDNA, IFN-γ, etc.	RIP1, RIP3, MLKL	Necrostatin-1 (Nec-1), GSK-843, GSK-872, GSK-840, TAK-632, NTB451, necrosulfonamide (NSA)
MPT-dependent necrosis	Mitochondrial swelling/rupture, permeability of IMM increase	Oxidative stress, ion overload	p53, CYPD, ANT, VDAC, F1FO ATP synthase	Cyclosporin A, sanglifehrin A, DS44170716, NIM-811, Debio025
Parthanatos	PARP1 overexpression, nuclear condensation	Oxidative stress, excitotoxic stress, genotoxic stress	PARP1, AIF	Olaparib, 4MF
Ferroptosis	GSH depletion, iron-dependent lipid ROS accumulation, system χ_c_^−^ inhibition	Erastin, RSL3/5, SAS, Sorafenib	System χ_c_^−^, GSH, GPX4	Ferrostatin-1, 16-86, liproxstatin
Oxytosis	GSH depletion, lipid ROS accumulation, system χ_c_^−^ inhibition, Ca^2+^ influx	Glutamate	System χ_c_^−^, GSH, GPX4, cGMP, AIF	C16, CdCl2, LaCl2, DIDS, sulfite
Pyroptosis	Caspase-1/caspase-11 activation, PM rupture	Bacterial infection, LPS	Caspase-1, caspase-11, GSDMD	AC-YVAD-CMK, Z-YVAD-FMK, VX-765
NETosis	Intracellular contents release, NET formation, chromatin unfolding	Bacterial infection, sterile inflammation, LPS	NETs, ROS, NOX, NE, MPO, histones	THIQs, Cl-amidine, Trolox, Tempo

Necroptosis is the most studied form of regulated necrosis. The first discovery of necroptosis was tracked to 1988, Laster et al. (1988) observed that tumor necrosis factor (TNF) triggered both apoptotic and necrotic death in various cell types. At that time, the term ‘necroptosis’ had not been proposed. For a decade, the TNF-induced cell death was considered to be the apoptosis. Until 1998, Vercammen et al. (1998) discovered that TNF-induced cell death was independent of caspases, the apoptosis-associated molecules. Meanwhile, Kawahara et al. (1998) reported that Fas-associated protein with death domain (FADD)-induced cell death was also independent of caspase-8 activation. These findings suggested that there was a novel type of cell death, which was different from apoptosis but similar to necrosis. In 2000, RIP1 was first verified as a crucial molecule that regulated Fas-induced necrosis in the caspase-8-inhibited cells (Holler et al., 2000). In 2005, the term ‘necroptosis’ was first proposed by Degterev et al. (2005) to describe Fas/TNF-induced nonapoptotic cell death, and they found an inhibitor of necroptosis, necrostain-1 (Nec-1). In 2009, several studies published that RIP3 kinase, the downstream target of RIP1, was required for necroptosis (Cho et al., 2009; He et al., 2009; Zhang et al., 2009). In 2012, MLKL, a necroptotic executor, was firstly discovered by Sun et al. (2012), which can be recruited and phosphorylated by RIP3. From then on, a number of studies were further focused on the downstream events that triggered by MLKL.

Necroptosis acts as an emerging form of programed necrosis. As for the mechanism, necroptosis differs from passive necrosis; it can be highly regulated by an intracellular protein platform just like apoptosis (Table 1). Typically, different cellular stimuli, including TNF, FAS ligand (also known as CD95L and APO-1L; Holler et al., 2000), TNF-related weak inducer of apoptosis (TWEAK; Wilson and Browning, 2002), TNF-related apoptosis-inducing ligand (TRAIL, also known as APO-2L), pathogen-associated molecular patterns, double-stranded RNA or DNA, interferons (IFNs), ATP depletion, ischemia‒reperfusion (I/R) injury, and some anti-cancer drugs (Huang et al., 2013), have been shown to induce necroptosis.

## MLKL

MLKL pseudokinase belongs to a class of proteins that are expressed as soluble polypeptides. It is composed of an N-terminal four-helix bundle domain (NB, amino acid residues 1‒125 in mouse and 1‒124 in human) and a C-terminal pseudokinase domain (psKD, residues 171‒464), which are connected by a two-helix linker or brace (Murphy et al., 2013). The NB domain is also called ‘killer’ domain (Petrie et al., 2017). The two brace helix not only tether the NB domain and the psKD, but also contribute to communicating psKD phosphorylation event to the NB domain and providing an interface for MLKL conformational changes and oligomerization (Davies et al., 2018). As for the psKD, it acts as a molecular switch that transforms between the activated and inactive conformations of MLKL (Murphy et al., 2013). In addition to control configuration change of MLKL, the psKD also functions as an adaptor for some proteins, such as well-characterized RIP3, heat-shock protein 90 (Hsp90), and the protein kinase co-chaperone CDC37 (Li et al., 2015; Jacobsen et al., 2016).

MLKL acts as a substrate of RIP3, another essential molecular component of necroptosis. MLKL can be phosphorylated by RIP3 at Thr-357 and Ser-358 in humans (Sun et al., 2012) and at Ser-345, Ser-347, and Thr-349 in mice (Murphy et al., 2013). These residues are present within the activation loop in the psKD of MLKL. The phosphorylation can induce a conformational change, releasing the NB domain, which is responsible for lipid engagement and membrane permeabilization. Two distinct MLKL isoforms, isoform 1 (MLKL1) and isoform 2 (MLKL2), have been reported, and this phenomenon is generated by alternative mRNA splicing. MLKL1 consists of an NB domain and a psKD, tethered by a two-helical brace region. MLKL2 has the same NB domain as MLKL1 but lacks the kinase core domain (Arnez et al., 2015).

A few MLKL inhibitors have been reported. Necrosulfonamide is an inhibitor of human MLKL, which was found to block necroptosis by targeting Cys-86 on MLKL (Sun et al., 2012). Compound TC13172, another MLKL inhibitor, induces covalent binding at Cys-86 on MLKL. GW806742X targets the psKD of MLKL (Hildebrand et al., 2014). These inhibitors may be promising drug candidates in a variety of diseases associated with MLKL-dependent necrosis.

## MLKL-dependent necrosis—necroptosis

### RIP1/RIP3/MLKL necrosome formation

**Figure 1:**
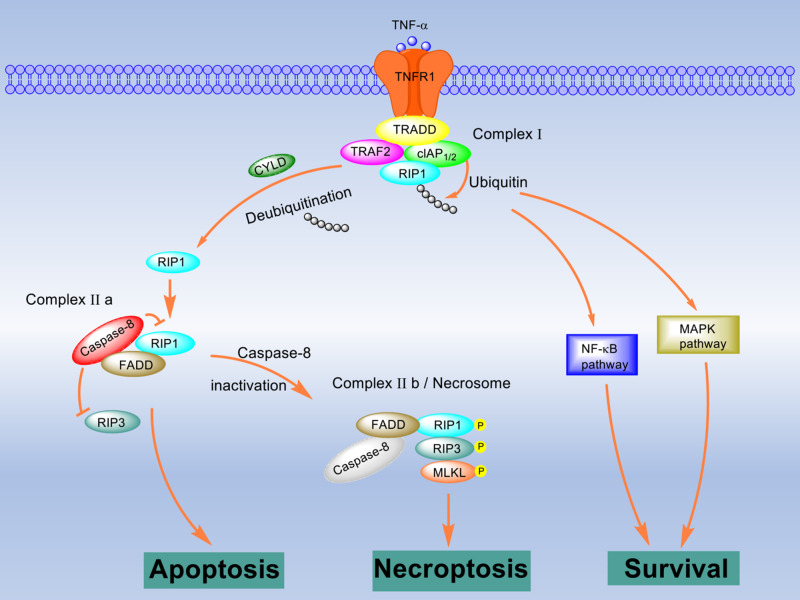
RIP1/RIP3/MLKL necrosome formation. TNF stimulation results in formation of complex I consisting of TRADD, RIP1, TRAF2, and cIAP_1/2_. In complex I, RIP1 is ubiquitinated by cIAPs, which leads to the activation of the NF-κB pathway or MAPK pathway and prevents cell death. Once RIP1 is deubiquitinated by CYLD, caspase-8, FADD, RIP1, and RIP3 are recruited and assembled to be complex IIa. In this complex, RIP3 and RIP1 are suppressed by active caspase-8, which results in apoptosis. When caspase-8 is inhibited or silenced, the necrosome (complex IIb) consisting of RIP1, RIP3, and MLKL is formed. Mutually direct or indirect phosphorylation of these molecules in the necrosome initiates necroptosis.

Among the identified necroptotic initiators, TNF is one of the best studied. Here, we take TNF as an instance to describe the signal pathway of the necrosome formation (Figure 1). TNF can be recognized by TNFR1 (de Almagro and Vucic, 2015). Then the activated TNFR1 forms a trimer and recruits TNFR1-associated death domain protein (TRADD), TNFR2-associated factor 2 (TRAF2), cellular inhibitor of apoptosis protein 1/2 (cIAP_1/2_), and serine/threonine poly-ubiquitinated RIP1 via their death domains to develop a molecular complex named complex I. In complex I, RIP1 is rapidly ubiquitinated by E3 ligases LUBAC and cIAP_1/2_ (de Almagro et al., 2015; Varfolomeev et al., 2015). The ubiquitinated RIP1 recruits NEMO and TAK1 to activate the NF-κB or MAPK pathways (Ea et al., 2006). These two pathways maintain the activation of caspase-8 and promote cell survival. If the ubiquitination is inhibited by antagonizing cIAP_1/2_, the cells are more sensitive to TNF-induced necroptosis (McComb et al., 2012). TNFR2, another receptor of TNF, does not mediate cell death alone but promotes TNFR1-mediated cell death by facilitating the degradation of TRAF2. CYLD, a K63-specific deubiquitinating enzyme, also mediates the deubiquitination of RIP1 to facilitate the formation of complex IIb, which is RIP1/RIP3/MLKL necrosome (Moquin et al., 2013). In the absence of CYLD, the formation of necrosome is significantly inhibited. The activation of RIP1 leads to the recruitment of RIP3, interacting through the RIP homology interaction motif (RHIM). After RIP3 is phosphorylated by RIP1 or itself on Ser-232, MLKL binds to it via the C-terminal kinase-like domain; in addition, MLKL is phosphorylated by RIP3. Thus, the RIP1/RIP3/MLKL necrosome is formed. Notably, FADD and caspase-8 are also detected in necrosome (Lin et al., 1999).

In addition to TNFR, TLRs such as TLR3 and TLR4 have also been demonstrated to trigger necrosome formation. When caspase-8 is inhibited, activated TLRs recruit the adaptor protein, Toll/IL-1 receptor domain-containing adaptor inducing IFN-β (TRIF). Then, the RHIM in TRIF interacts with RIP1 and RIP3 (He et al., 2011). Remarkably, in the absence of RIP1, TRIF-dependent necroptosis can also proceed (Kaiser et al., 2013).

### Downstream events after MLKL activation

**Figure 2:**
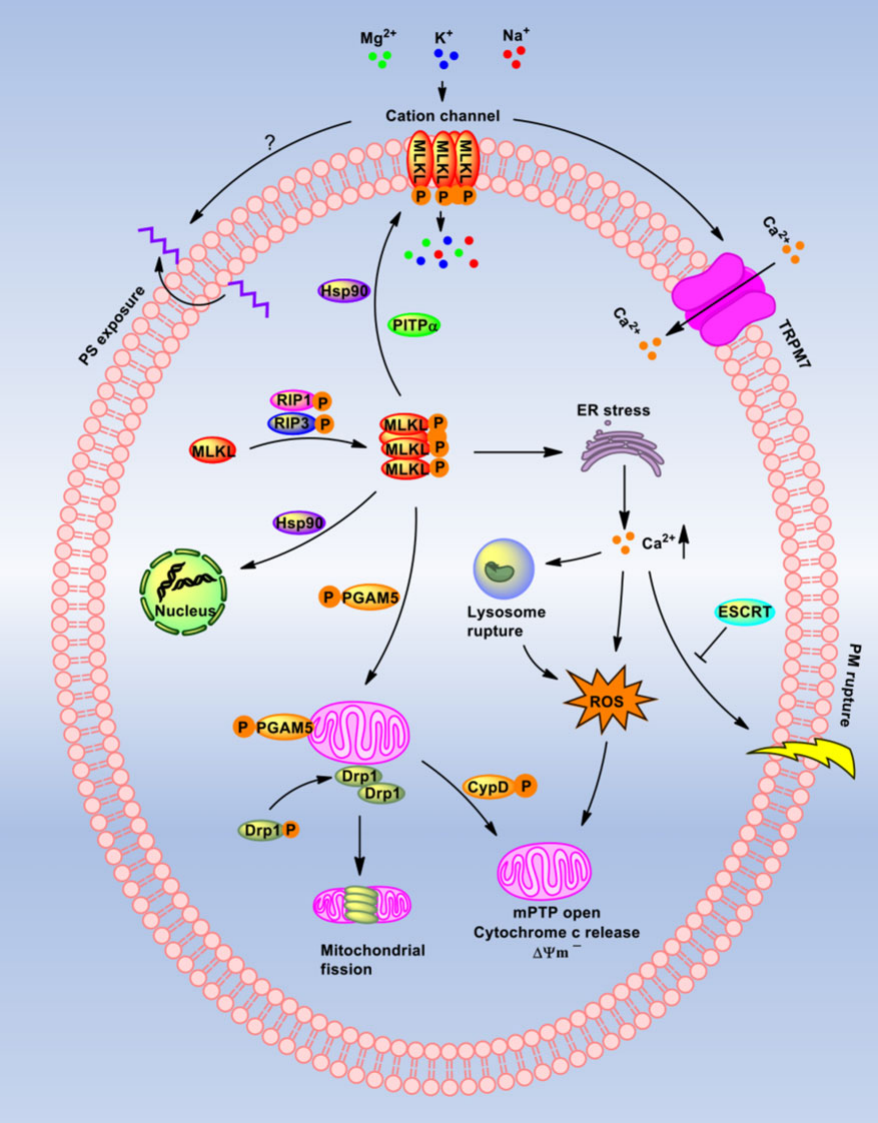
Downstream events of MLKL-mediated necroptosis. MLKL is phosphorylated and oligomerized by RIP3. One part of MLKL oligomers translocate to the nucleus and the other to the PM, and the translocation is assisted by Hsp90 or PITPα. The oligomers in PM mediate a cation channel formation to allow extracellular ion (Mg^2+^, K^+^, and Na^+^) influx. PS exposure to extramembrane to release ‘find/eat-me’ signals is also mediated by oligomers. Besides, TRPM7 is open and allows Ca^2+^ influx. The oligomers initiate ER stress to produce ROS and release Ca^2+^. The high level of calcium in the cytoplasm may contribute to lysosomal dysfunction, ROS production, and PM rupture, which can be inhibited by ESCRT. Elevated ROS stimulates mPTP opening, mitochondrial membrane potential change, and cytochrome c releasing. Moreover, PGAM5 on mitochondria binds to the necrosome, which further recruits and activates drp1 or CypD to mediate mitochondrial fission or mPTP opening. All the mentioned evens could directly or indirectly induce necroptotic cell death.

The downstream mechanism of MLKL in necroptosis is very complicated and is tissue- and cell type-specific. Roughly, several events that follow the MLKL phosphorylation are summarized (Figure 2).

At first, it has been reported that phosphorylated MLKL can form oligomers in necrosome, which are proposed to be trimers (Cai et al., 2014), tetramers (Petrie et al., 2018), hexamers (Wang et al., 2014), or octamers (Huang et al., 2017). In polymers, the monomeric MLKL is connected by a disulfide bond regulated by the thiol oxidoreductase thioredoxin-1 (Reynoso et al., 2017). The formed oligomers move from the cytoplasm to the cell membrane and insert deeply into the membrane bilayer by binding to phosphatidylinositol lipids or cardiolipin. Then, the oligomers directly disrupt membrane integrity, consequently resulting in cell death. Interestingly, a small number of MLKL is translocated to the nucleus after activation, which might also facilitate cell death (Yoon et al., 2016).

In necroptotic signaling, the MLKL oligomerization and membrane translocation are two essential events, and the mechanisms are still unclear. Hsp90, a well-known chaperone, has been identified to regulate MLKL oligomerization and/or membrane translocation by affecting the phosphorylation-induced conformational changes in MLKL (Jacobsen et al., 2016). Phosphatidylinositol transfer protein alpha (PITPα), which is responsible for the transfer of phosphatidylinositol between membranes, has also been reported to facilitate MLKL oligomerization and membrane translocation by interacting with the MLKL N-terminal on its sixth helix and the preceding loop (Jing et al., 2018).

Ion influx occurs immediately upon MLKL oligomerization and translocation, hence disrupting osmotic homeostasis. The six helices (H1‒H6) in MLKL N-terminal domain form a cation channel in the PM, which is permeable to Mg^2+^, Na^+^, and K^+^ but not Ca^2+^ (Xia et al., 2016). However, a variety of studies have reported that Ca^2+^ influx is necessary for MLKL-mediated necroptosis (Liao et al., 2017). On the one hand, the study suggested that the increased Ca^2+^ in the cytoplasm is from the intracellular Ca^2+^ pool, such as endoplasmic reticulum (ER), instead of the culture media (Gong et al., 2017a). In HT29 cells, transient receptor potential melastatin related 7 (TRPM7; Faouzi et al., 2017), a well-known non-voltage-sensitive calcium channel, has been identified to be a downstream component of MLKL and is responsible for the calcium influx and subsequent PM damage (Cai et al., 2014). How Ca^2+^ influx mediates cell necroptosis remains controversial. First, intracellular calcium overload not only causes PM damage, but also in cardiomyocytes leads to xanthine oxidase expression and further activates cellular reactive oxygen species (ROS), which induces the mitochondrial permeability transition pore (mPTP) opening and necroptosis (Zhu et al., 2018). Moreover, increased intracellular Ca^2+^ and the activation of Ca^2+^-dependent enzymes, calpains, lead to lysosomal membrane permeabilization, which allows cathepsin B and cathepsin D releasing to the cytosol. These enzymes further result in ROS production, depolarization of the mitochondrial membrane, and cytochrome c releasing (Zhao et al., 2003; Yashin et al., 2016).

Phosphatidylserine (PS) exposure on the outer leaflet of the PM is another event, which can occur within 5 min of MLKL activation (Zargarian et al., 2017). Still now, the key molecules responsible for PS externalization during necroptosis have not yet been discovered. However, the exposed PS would provide the ‘find-me’ and ‘eat-me’ signals to dendritic cells and macrophages, which is also called ‘efferocytosis’ (Elliott et al., 2017). The engulfed necroptotic cells keep the PM integrity, suggesting that there must be a mechanism for cells to repair damage and maintain survival. Some studies suggest that the ‘bubbles’, which are largely broken and do not contain cytosolic proteins, occur at the sites where the NB domain of MLKL targets the PM, and these broken bubbles release from the intact cells to sustain PM integrity during necroptosis (Gong et al., 2017b). In this repair mechanism, the endosomal sorting complexes required for transport (ESCRT) components, including CHMP2A, CHMP4B, VPS4, TSG101, and IST1, play the crucial roles. Silencing these components can prevent bubbles from formation and accelerate necroptosis (Gong et al., 2017b). Interestingly, Ca^2+^ has been identified to be required for ESCRT-mediated PM repair (Scheffer et al., 2014), and depletion of extracellular Ca^2+^ prevents the formation of PM bubbles. Thus, during necroptosis, Ca^2+^ regulates both MLKL-triggered pore-forming and repair processes.

Phosphoglycerate mutase family member 5 (PGAM5), a mitochondrial phosphatase with homology to the family of phosphoglycerate mutases but lacking similar enzymatic function, is the anchor of RIP1/RIP3/MLKL necrosome on mitochondria (Wang et al., 2012). After RIP1/RIP3/MLKL necrosome formation, the two splicing variants of PGAM5, PGAM5L and PGAM5S, sequentially bind to the necrosome. Activated PGAM5 recruits and activates the dynamin-related protein 1 (drp-1), a large GTPase associated with mitochondrial fission (Smirnova et al., 1998). Moreover, in the I/R injury model, the activation of PGAM5 can also promote cyclophilin D (CypD) phosphorylation on serine/threonine residues (Alam et al., 2015). Then, the phosphorylated CypD binds to the inner mitochondrial membrane, which facilitates the mPTP opening and further triggers cellular necroptosis (Crompton et al., 1998).

### MLKL-dependent necroptosis in diseases

A large number of studies have proved that necroptosis plays a vital role in the pathogenesis of various diseases. For example, cardiomyocytes’ necroptosis could induce several heart diseases, including myocardial infarction (MI), I/R injury, heart failure (HF), and cardiomyopathy (Zhu and Sun, 2018). Atherosclerosis is another disease associated with necroptosis. The expression of both RIP3 and MLKL demonstrates a significant increase in atherosclerotic plaques compared to normal arteries (Karunakaran et al., 2016). Besides, numerous studies have shown that MLKL-dependent necroptosis is critical in the pathogenesis of brain diseases, especially ischemic brain injury and neurodegenerative diseases (Zhang et al., 2017; Xu et al., 2018a). Necroptosis has also been implicated in acute kidney injury (AKI) and chronic renal disease (Imamura et al., 2018). Furthermore, a variety of inflammatory diseases, including trauma (You et al., 2008), colitis (Pierdomenico et al., 2014), pancreatitis (He et al., 2009), and sepsis (Vucur et al., 2018), are associated with necroptosis. In addition, RIP3-dependent necroptosis is the main cause of embryonic lethality in caspase-8-deficient mice (Kaiser et al., 2011).

Accumulating evidence indicates that downregulation and mutations in the key necroptotic regulators, including RIP1, RIP3, and MLKL, are found in a variety of cancers by allowing tumor cells to evade necroptosis and possibly immune surveillance (Chen et al., 2016). Thus, the therapy based on necroptosis might be a novel strategy for anti-tumor treatment.

## MLKL-independent necrosis

### MLKL-independent necroptosis

The calmodulin-dependent protein kinase II (CaMKII), a serine‒threonine kinase that is abundant in the myocardium and other excitable tissues, was identified as another potential substrate of RIP3. RIP3 can directly mediate CaMKII, instead of the constitution of the RIP3/MLKL complex, to induce necroptosis under severe cardiac pathological conditions, including MI, I/R injury, and HF (Zhang et al., 2016).

RIP3-induced activation of CaMKII, via phosphorylation at Thr-287 and oxidation at Met-281/282 or both, triggers the opening of mPTP leading to necroptosis (Joiner et al., 2012). Moreover, CaMKII can also regulate multiple ion channels after activation, including the L-type Ca^2+^ channel subunit CaV1.2 β (Koval et al., 2010), the Na^+^ channel NaV1.5 (Maltsev et al., 2008), and the K^+^ channel Kv4.3 (Sergeant et al., 2005). Activation of these ion channels may result in an influx of extracellular ions and eventually induce PM rupture and cell necroptosis (Grootjans et al., 2017).

### Na^+^/K^+^-ATPase-mediated necrosis

**Figure 3:**
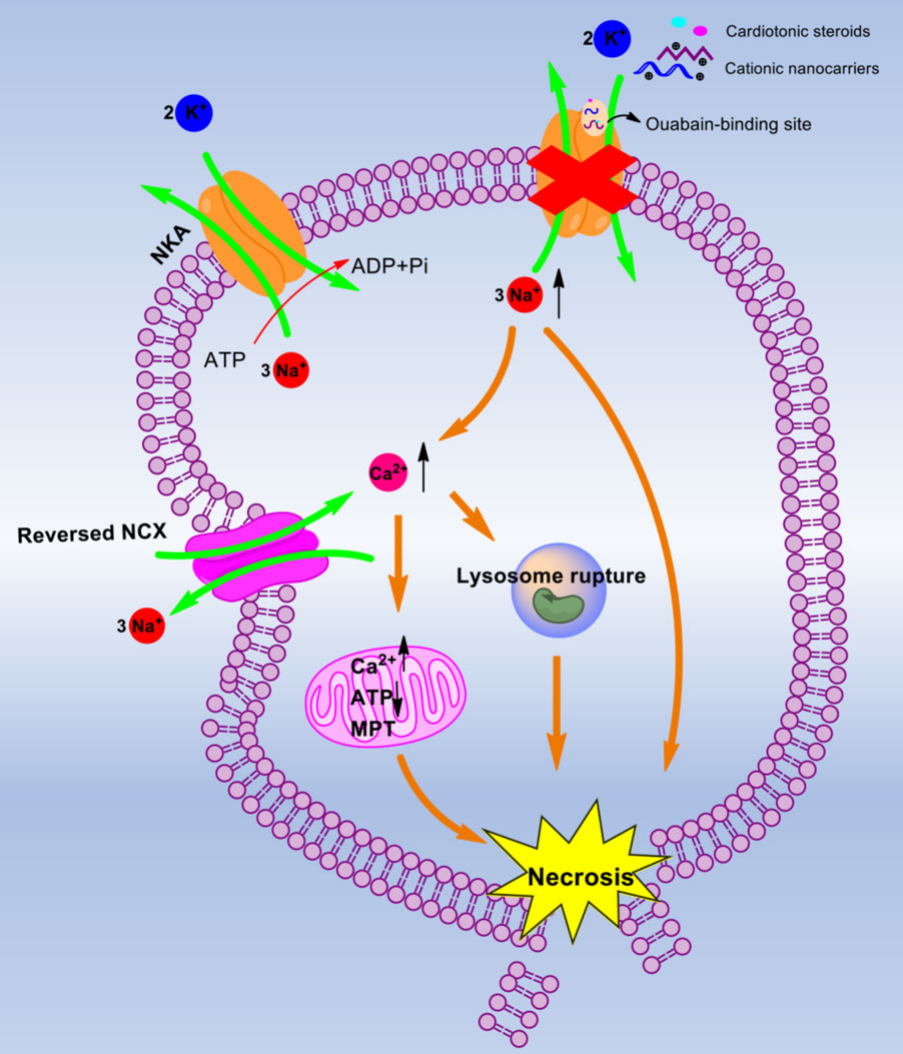
The mechanism of NKA-mediated necrosis. Cationic nanocarriers and cardiotonic steroids bind to the ouabain-binding site and inhibit NKA. This inhibiting effect leads to Na^+^ overload in the cytosol. The increased Na^+^ directly mediates necrosis or promotes Ca^2+^ uptake through the reversed NCX. The excess Ca^2+^ influx triggers lysosome rupture and mitochondrial Ca^2+^ overload, which leads to MPT, ATP depletion, and eventually necrosis.

Na^+^/K^+^-ATPase (NKA), a sodium pump existing in the PM, transports three Na^+^ out of the cell and two K^+^ into the cell with single ATP consumption (Geering, 2008). When the PM suffers from stimulation, this enzyme is highly vulnerable and easily causes disorders of cellular ions. For instance, cationic nanocarriers (e.g. DOTAP, PEI, and chitosan) bind to NKA in the PM at the ouabain-binding site, which further leads to intracellular Na^+^ overload, cellular osmotic pressure change, and eventually rapid cell necrosis (Wei et al., 2015). Similarly, cationic cell-penetrating peptides induce cell swelling via impairment of NKA and Na^+^ overload (Qiu et al., 2017). Besides, cardiotonic steroids including digoxin and ouabain specifically inhibit NKA, increase intracellular Na^+^ concentration, and cause cell swelling (Cui and Xie, 2017). The increase in cell volume is a key character of necrosis, and NKA plays a crucial role in regulating cell volume. According to Gibbs–Donnan equilibria, soon after NKA is blocked, the membrane potential rises and reaches the spiking threshold (Dijkstra et al., 2016). The cell volume increases without bound, eventually leading to cell lysis. This also explains the reason that cationic nanocarriers induce cell depolarization before the earliest cell necrosis (Wei et al., 2015). Note that the osmotic imbalance and cytotoxic edema result from the disordered ion permeabilities (Takeuchi et al., 2006). The impaired NKA induces the disruption of K^+^, Na^+^, Cl^−^, and Ca^2+^ homeostasis and hybrid death. Then, intracellular K^+^ loss induces apoptotic cell death with caspase activation, cytochrome c release, DNA degradation, and cell shrinkage (Yu, 2003). Meanwhile, the accumulation of intracellular of Na^+^, as well as the membrane depolarization, promotes a Na^+^-dependent Ca^2+^ uptake through various channels such as the reversed Na^+^/Ca^2+^ exchanger (NCX) (Tian and Xie, 2008) or voltage-sensitive Na^+^ channels (Martinez-Sanchez et al., 2004). Numerous studies have shown that increased intracellular Na^+^ and Ca^2+^ lead to cytotoxic cell swelling, PM rupture, and the ensuing necrosis (Song and Yu, 2014). Na^+^ overload is usually considered to be a major factor for necrosis (Zong and Thompson, 2006), stimulating cells to swell up and die much faster than those whose NKA is inhibited by ouabain or incubated with Na^+^-free medium (Wei et al., 2015). On the other hand, excess Ca^2+^ influx elevates the intracellular Ca^2+^ concentration, which has been suggested to mediate necrosis by rupturing lysosomal membranes and then releasing lysosomal proteases (Yamashima, 2004). Furthermore, cytosolic Ca^2+^ overload triggers mitochondrial Ca^2+^ overload, which leads to mitochondrial permeability transition (MPT), ATP depletion, and apoptosis-inducing factor (AIF) release (Nakayama et al., 2007; Figure 3).

### MPT-dependent necrosis

MPT-dependent necrosis is another MLKL-independent programed necrosis, which is characterized by an abrupt increase in the permeability of the inner mitochondrial membrane to small solutes, collapse of mitochondrial membrane potential, and mitochondrial swelling or rupture (Kroemer et al., 2007). The identified triggers of MPT-dependent necrosis include oxidative stress, ion overload (e.g. H^+^, Ca^2+^, Mg^2+^, and phosphates), adenine nucleotides, ubiquinones, etc. (Javadov and Kuznetsov, 2013). In response to oxidative stress, p53 accumulates in the mitochondrial matrix, interacts with CypD and then leads to mPTP opening (Vaseva et al., 2012). Besides, adenine nucleotide translocase, voltage-dependent anion channel (VDAC), phosphate carrier, translocator protein, metalloprotease spastic paraplegia 7, and F1FO ATP synthase are also reported to contribute to MPT-dependent necrosis (Briston et al., 2019). However, the precise mechanisms for the activation of MPT-dependent necrosis remain obscure. MPT-dependent necrosis has also been implicated in the pathogenesis of I/R injury in the heart (Zhu and Sun, 2018) and liver (Zhang and Lemasters, 2013) and neurodegenerative diseases (Briston et al., 2019).

### Parthanatos

The term ‘parthanatos’ was first coined by Andrabi et al. (2008) to describe a poly(ADP-ribose) (PAR) polymerase 1 (PARP1)-mediated necrosis. PARP1 belongs to the family of PARP proteins, which transfer ADP-ribose groups from NAD^+^ to multiple substrates and then control cellular processes (Gibson and Kraus, 2012). PARP1 can be activated by DNA breaks and recruited to the site of DNA damage to perform DNA repair (Satoh and Lindahl, 1992). Although PARP1 activation maintains cellular homeostasis, hyperactivated PARP1 results in parthanatos (David et al., 2009). The hyperactivated PARP1 triggers NAD^+^ (consequently ATP) depletion and inhibits mitochondrial ATP synthesis in cells. In particular, the binding of PAR to AIF is required for parthanatos (Wang et al., 2011). Hyperactivated PARP1 leads truncated AIF to release from the cytosolic side of the mitochondrial outer membrane (Yu et al., 2006; Wang et al., 2011). The released AIF is transferred to the nucleus, where it binds to flavin adenine dinucleotide (FAD) or DNA and acts as an endonuclease to promote chromatin degradation (Hangen et al., 2010). Interestingly, in hydrogen peroxide (H_2_O_2_)-induced parthanatos of retinal pigment epithelium, translocation of AIF was not observed (Jang et al., 2017). Thus, the molecular machinery of parthanatos is still unclear. Parthanatos is seen in many diseases including I/R injury, stroke, heart attack, Parkinson’s disease, and diabetes (Wang et al., 2010).

### Ferroptosis

Yang and Stockwell (2008) discovered that three small molecules (erastin, RSL3, and RSL5) triggered an iron-dependent cell death, also called ‘ferroptosis’, in RAS-transformed cancer cells. Ferroptosis is triggered by the inhibition of the cystine (Cys)/glutamate (Glu) antiporter termed system χ_c_^−^, which imports extracellular L-Cys in exchange for intracellular L-Glu (Dixon et al., 2012). Once the system is inhibited, the uptake of Cys is blocked and Cys-dependent glutathione (GSH) synthesis will be restrained, which both contribute to the accumulation of toxic lipid ROS and cell death (Dixon et al., 2012). Activation of p53 has been suggested to induce ferroptosis by directly inhibiting the transcription of SLC7A11, a key component of system χ_c_^−^ (Jiang et al., 2015). Glutathione peroxidase 4 (GPX4), a GSH-dependent enzyme that reduces lipid hydroperoxides (L-OOH) to lipid alcohols (L-OH), effectively inhibits ferroptosis through regulating GSH level and ROS production (Friedmann Angeli et al., 2014). In ferroptosis, the depletion of polyunsaturated fatty acids (PUFAs) is also observed to respond to Cys depletion and GPX4 inactivation (Friedmann Angeli et al., 2014). PUFAs are susceptible to oxidation, leading to the formation of L-OOH (Cheng and Li, 2007). In the presence of iron, L-OOH forms toxic lipid radicals such as alkoxy radical L-O^−^. The lipid radicals can obtain protons from adjacent PUFAs, initiating a new round of lipid oxidation (Magtanong and Dixon, 2018). Lipid ROS is formed upon the PUFA chains in the membrane lipids. Thus, the iron chelators may block ferroptosis by preventing iron to donate electrons in the redox (Dixon and Stockwell, 2014). Ferroptosis has a crucial role in anti-tumor therapies, brain diseases, I/R injury, and AKI (Tang et al., 2018).

### Pyroptosis

The term ‘pyroptosis’ was initially introduced by Brennan and Cookson (2000) to defined the caspase-1-dependent death of macrophages infected by *Salmonella enterica* subsp. *enterica serova Typhimurium*. Notably, pyroptosis is not restricted to caspase-1-dependent bacterial infections and macrophage death (Kepp et al., 2010). In later studies, it has been reported that caspase-11 can also trigger macrophage pyroptosis in response to various Gram-negative bacterial infections (Kayagaki et al., 2011). Morphologically, the pyroptotic cells undergo cell swelling, PM rupture, cytoplasmic content release, and nucleus condensation. In caspase-1-dependent pyroptosis, caspase-1 is activated by canonical inflammasomes, including NLRP1, NLRP3, NLRC4, and AIM2 (Xu et al., 2018b), and the activated caspase-1 mediates the formation of PM pores with a diameter of 1.1–2.4 nm, which leads to osmotic cell lysis (Fink and Cookson, 2006). Gasdermin D (GSDMD) protein is recently identified as a key pyroptosis substrate of caspases (Shi et al., 2015). Activated caspase-1 and caspase-11 can efficiently cleave GSDMD into two parts, N domain and C domain. The N domain of GSDMD binds to the PM and oligomerizes to form membrane pores with an inner diameter of 10–14 nm (Ding et al., 2016). Of note, GSDMD-deficient cells can still die, which is likely due to caspase-1 cleavage of caspase-3/7 (Lamkanfi et al., 2008).

Pathogenic microbes and viruses can induce pyroptosis to control infection. Pyroptosis was also reported to play an important role in other diseases, including inflammatory bowel disease (Yuan et al., 2018), diabetic retinopathy (Homme et al., 2018), liver fibrosis, and hepatocellular carcinoma (Guo et al., 2019).

### Oxytosis

Oxytosis or excitotoxicity is a neuronal cell death triggered by an excess of the neurotransmitter Glu (Tan et al., 2001). Similar to ferroptosis, system χ_c_^−^ inhibition, GSH depletion, and lipid ROS accumulation are all covered in oxytosis (Lewerenz et al., 2018). However, the exact degree of overlap between oxytosis and ferroptosis is still murky. GSH depletion in oxytosis activates 12-lipoxygenase (LOX12) and LOX15. Both LOX12 and LOX15 integrate into the membranes to initiate ROS production and Bid activation, which induces AIF released from mitochondria and an increase in cyclic guanosine monophosphate (cGMP) (van Leyen et al., 1998; Landshamer et al., 2008). cGMP modulates store-operated calcium entry and opens cGMP-gated channels, allowing calcium influx (Henke et al., 2013). The ‘calpain–cathepsin cascade’ occurs downstream of the calcium wave and is involved in the activation of calpains by calcium (Elphick et al., 2008), which induces lysosomal membrane permeabilization to induce cell death. VDAC1 plays a crucial role in Glu-mediated oxytosis, and the inhibitor of VDAC1 could significantly reduce ROS production and improve cell survival, which may also be due to the inhibition of [Ca^2+^]_m_ uptake (Nagakannan et al., 2019). However, the precise mechanisms of oxytosis and the difference between ferroptosis and oxytosis need further investigation. Similar to ferroptosis, oxytosis is mainly associated with nerve cell death in the development of the central nervous system, neurodegenerative diseases, and neurotrauma (Tan et al., 2001).

### NETosis

NETosis is a self-sacrificing strategy for neutrophils that releases neutrophil extracellular traps (NETs), which are composed of histones and chromatin decorated with antimicrobial proteins (Urban et al., 2009), to capture and kill pathogens (Brinkmann et al., 2004). The NETosis is characterized by intracellular content release and chromatin unfolding. Apart from neutrophils, this kind of cell death is also discovered in eosinophils and mast cells, which is generalized termed ‘ETosis’ (Wartha and Henriquesnormark, 2008). The molecular mechanisms underlying NETosis are still unclear. Several central proteins in NETs including neutrophil elastase (NE), myeloperoxidase (MPO), and the NADPH oxidase (NOX) complex have been well characterized (Brinkmann et al., 2004; Fuchs et al., 2007). During NETosis, the NOX complex is activated by phorbol 12-myristate 13-acetate and produces superoxide anions ($O_{2}^{-}$) that are further converted to H_2_O_2_ (Martinez et al., 2017). H_2_O_2_ is a substrate of MPO and induces the release of NE. The released NE subsequently moves to the nucleus to induce histone degradation and lead to DNA concentration. MPO also moves to the nucleus and decondenses chromatin (Metzler et al., 2014). After nuclear and PM rupture, the decondensed chromatin, mixed with NE, MPO, and other proteins, expels to extracellular space. NE, MPO, histones, and other mixed proteins all play key roles in the antibacterial function of NETs (Urban et al., 2006). The NET formation in neutrophils is lethal to itself. NETosis is involved in various diseases, including autoimmune diseases, cardiovascular diseases, infectious diseases, and diabetes (Gupta and Kaplan, 2016; Berthelot et al., 2017).

## Concluding remarks

During the exploration of the complexity of necrosis, several emerging regulated necrosis are identified. In this review, we focused on the mechanism for the initiation and execution of MLKL-dependent necroptosis. When necroptosis is evoked by the death-receptor ligands or other stimuli, a series of molecular proteins are recruited and activated. Among these effective proteins, RIP1, RIP3, and MLKL act as the core machinery in the classical necroptotic pathway. The phosphorylated and oligomerized MLKL is considered to be an executioner, which can translocate to the nucleus and PM and further lead to membrane rupture, mitochondrial dysfunction, or other downstream events, which mediate necroptotic cell death. However, in some special cases, MLKL is not invariably involved in the necroptotic pathway. CaMK II, an emerging downstream target of RIP3, could also trigger necroptosis. In addition to necroptosis, the mechanisms of other types of necrosis including NKA-mediated necrosis, pyroptosis, ferroptosis, parthanatos, oxytosis, and NETosis are also summarized. Generally, MLKL-dependent and MLKL-independent necrosis can be seen in multiple tissues or organs, which may be related to the pathophysiology of multiple diseases, such as heart disease, atherosclerosis, brain disease, infection, and inflammation.

In future studies, the efforts should be paid on the following three aspects. First, the exact mechanism for each type of regulated necrosis and connection between them should be further investigated. For MLKL-dependent necrosis, how the MLKL oligomers located in the nucleus influence cell death, how MLKL transduces death signals to its downstream effectors, and how the MLKL oligomers cause membrane rupture need to be expounded in detail. Second, scientists should recognize new regulators for necrosis or discover new types of regulated necrosis. Third, the contribution of necrosis to each pathophysiological model and the potential therapeutic targets for these diseases must be clearly described. Besides, novel selective inhibitors targeting necrosis should be developed.

Although this review summarized the reported molecular events participating in necrosis, the unabridged mechanisms are still unclear. With the advanced biological detection technology appearing, the mystery masks on necroptosis would be unveiled.
